# Medical Nursing

**Published:** 1895-03

**Authors:** 


*Medical Nursing.* By the late JAMES ANDERSON, M.D. Edited by ETHEL F. LAMPORT. Pp. xii., 184. Second Edition. London: H. K. LEWIS. 1895.

That two editions of this book should have been issued in three months suffices to show that the late Dr. J. Anderson had already established a reputation as a teacher and physician. Of these lectures on nursing, only the introductory one was fully written out by the author; the others have been put together by Miss Lamport from the brief notes of the author himself and from others taken down at lecture by some members of his audience. This probably accounts for the rather incoherent character of the composition and lack of order in arrangement. It must be borne in mind that these lectures were but the concluding course of a series, and that this book, therefore, is not a complete manual of nursing; but it will be useful to nurses of some experience who have other text books. In the second edition some small improvements have been made, although some errors remain uncorrected. A sympathetic notice of the author by the late Sir Andrew Clark prefaces the body of the work.

